# Recent Applications of Advanced Atomic Force Microscopy in Polymer Science: A Review

**DOI:** 10.3390/polym12051142

**Published:** 2020-05-17

**Authors:** Phuong Nguyen-Tri, Payman Ghassemi, Pascal Carriere, Sonil Nanda, Aymen Amine Assadi, Dinh Duc Nguyen

**Affiliations:** 1 Institute of Research and Development, Duy Tan University, Da Nang 550000, Vietnam; 2 Département de Chimie, Biochimie et Physique, Université du Québec à Trois-Rivières (UQTR), Trois-Rivières, QC G8Z 4M3, Canada; payman.ghassemi3@gmail.com; 3 Laboratoire MAPIEM (EA 4323), Matériaux Polymères Interfaces Environnement Marin, Université de Toulon, CEDEX 9, 83041 Toulon, France; pascal.carriere@univ-tln.fr; 4 Department of Chemical and Biological Engineering, University of Saskatchewan, Saskatoon, SK S7N 5A2, Canada; sonil.nanda@usask.ca; 5 ENSCR—Institut des Sciences Chimiques de Rennes (ISCR)—UMR CNRS 6226, Univ Rennes, 35700 Rennes, France; aymen.assadi@ensc-rennes.fr; 6 Faculty of Environmental and Food Engineering, Nguyen Tat Thanh University, 300A Nguyen Tat Thanh, District 4, Ho Chi Minh City 755414, Vietnam; nguyensyduc@gmail.com; 7 Department of Environmental Energy Engineering, Kyonggi University, Suwon 16227, Korea

**Keywords:** AFM-IR, polymers, polymer composites, blends, polymer aging, nanoscale characterization, nanoscale characterization

## Abstract

Atomic force microscopy (AFM) has been extensively used for the nanoscale characterization of polymeric materials. The coupling of AFM with infrared spectroscope (AFM-IR) provides another advantage to the chemical analyses and thus helps to shed light upon the study of polymers. This paper reviews some recent progress in the application of AFM and AFM-IR in polymer science. We describe the principle of AFM-IR and the recent improvements to enhance its resolution. We also discuss the latest progress in the use of AFM-IR as a super-resolution correlated scanned-probe infrared spectroscopy for the chemical characterization of polymer materials dealing with polymer composites, polymer blends, multilayers, and biopolymers. To highlight the advantages of AFM-IR, we report several results in studying the crystallization of both miscible and immiscible blends as well as polymer aging. Finally, we demonstrate how this novel technique can be used to determine phase separation, spherulitic structure, and crystallization mechanisms at nanoscales, which has never been achieved before. The review also discusses future trends in the use of AFM-IR in polymer materials, especially in polymer thin film investigation.

## 1. Introduction

Fourier-transform infrared spectroscopy (FT-IR) is a popular spectroscopic technique used for the characterization and identification of numerous materials, especially for the polymers, biomaterials and life sciences [1,2,3,4,5]. However, the main drawback of the FT-IR method relates to its low resolution (under 20 µm). Optical microscopy is also a powerful method to study the polymer morphology, but remains limited by the diffraction limit, which is proportional to the wavelength (λ) of light:

(1) $$\Delta x\gg 0.61\cdot \frac{l}{n\cdot \sin\theta }$$

where (Δx) is the distance between the two samples and (n) represents the refractive index. Moreover, θ denotes the objective’s approval angle. The value of “n·sinθ” is generally referred to as the objective’s numerical aperture. Equation is obtained from Rayleigh’s criterion according to which two points can be determined provided the center of one Airy disk correlates with the minimum value of the next Airy pattern [6,7,8]. According to Equation, the lateral resolution of IR-based microscopes is often smaller than that of visible light-based microscopes because of the longer wavelength of the mid-IR light (λ = 2.5–10 μm) [9].

The addition of several special devices like attenuated total reflection crystals in an infrared (IR) spectroscopy (ATR-FTIR) allows the analysis of penetration depth into the sample between 0.5 and 2 µm [10]. Nevertheless, ATR-FTIR can be only performed on a spatial resolution approaching the wavelength with a sample thickness of 3–10 µm [10,11,12,13,14,15]. In contrast, the atomic force microscope (AFM) [16] is considered a useful tool for nanoscale measurement in polymer science and engineering [17,18,19], especially for studying complex materials [20,21,22,23,24,25,26,27,28,29]. Various newer discoveries have been reported in the literature with this nanoscale imaging technique [30,31,32,33,34,35,36,37,38] for structures and polymer crystallization. However, the chemical characterization of materials is limited mostly to AFM. While AFM provides information on a material’s morphology, friction behavior and adhesion at the nanoscale, it cannot provide the information on chemical composition or distribution of the functional groups of the polymer materials.

The idea of chemical modification of the probes was brought in to make them sensitive to particular molecular interactions to overcome this inherent limitation of the AFM [39]. Chemical Force Microscopy (CFM) was used for the separation between hydrophilic and hydrophobic regions [40] and interactions between particular functional groups, including such polar molecules, and acid-base interactions with resolution nanometer scales [41]. CFM uses a functionalized AFM tip with a high-quality probe. Low-quality tips can result in poor imaging with various artifacts [42]. Some important considerations include the material of the probe, nominal tip length, spring constant, and resonant frequency that suits the object [43]. More recently, a new nanoscale spectroscopy, so-called photo-induced force microscopy (PiFM) has been developed to study the gradient of optical forces during the light absorption of molecular vibrational resonances, which are applied to a high sensitive AFM.

Such recent attempts effectively break the diffraction limit of light and provide vibrational spectroscopy with a nanoscale spatial resolution that is a crucial tool for characterizing novel energy materials [44]. The coupling of Raman spectroscopic and an AFM is also reported, which is called nano-Raman or AFM-Raman [45,46,47,48,49,50,51,52,53,54]. Other techniques such as atomic force microscopy-infrared spectroscopy (AFM-IR) and infrared scattering-type scanning near-field optical microscopy (IR s-SNOM) [55,56,57,58] have also been developed to overcome these disadvantages. AFM-IR is capable of measuring and mapping the local chemical composition, which is usually below the diffraction limit to provide nanoscale images as the traditional AFM [55]. This method has been considered as an important development in sub-micrometer spectroscopies and chemical imaging. The use of this technique has shed light on many assumptions and has provided new mechanisms in the investigation of polymer materials [56,57].

Nowadays, AFM-IR is being applied for analyzing various systems including polymer blends [58,59], polymer composites [60,61,62,63], multilayer films [64], polymer thin films [65,66] and in life sciences [46,48,67,68,69] and other materials [55]. The AFM-IR can provide both a nanoscale AFM image and a high-resolution chemical spectrum on the sample within selected regions, thus helping to identify or qualitatively analyze many complex systems. Although there have been several review articles on the use of AFM and AFM-IR [55,70,71,72] in material science, there is a lack of literature regarding the usefulness of this technique in polymer science. Moreover, the information on the recent applications of AFM-IR in polymer crystallization is also lacking. The goal of this review article is to provide to readers the latest advancements in the use of AFM and AFM-IR techniques in structurally characterizing polymers blends, polymer composites and multilayers, especially on polymer aging as well as on crystallization studies.

## 2. The Principle of AFM and AFM-IR

AFM is a microscope used for the characterization of various processes at the nanoscale that involves investigations of quantitative single molecules [33]. AFM is an exceptional tool for studying the application of temporal shifts in the morphology of polymers. The advancement of devices operating at elevated temperatures has allowed observing in-situ of the polymer crystallization by following the crystal and spherulite growth as a function of time [73]. As an important tool, AFM was commonly used in the field of polymer crystallization. Generally, the effect of the AFM tip throughout the scan should be diminished to truly reflect the crystal morphology and crystallization mechanism [74]. It is a high-resolution tool usually with a resolution of sub-10 nm features [75,76].

AFM does not require sample staining or metal coating. Hence, it is straightforward to prepare the specimen. In many cases, it is also non-destructive. It makes images to be captured whereas a process such as melting or crystal growth occurs, providing lamellar or sub-lamellar resolution time-resolved data [77,78,79,80]. An opportunity for AFM is to study the crystallization of semi-crystalline polymers due to its enable to observe crystal melting, crystal growth and lamellar reorganizations and assembly, to see how spherulites grow, and how crystallization conditions (temperature, chemical composition and sample thickness) influence crystallization kinetics [80].

A pyramidal tip attached to a cantilever (typically 100–400 μm and made of silicon or silicon nitride) is an important sensor of AFM. The resolution of an image is determined by the geometry of the apex of the pyramidal tip. The sensor operated in two modes, i.e., contact mode and non-contact or dynamic mode. In the contact mode, the tip comes in direct contact with the surface of the sample, whereas in the dynamic mode, the tip oscillates to interact with the sample surface. The cantilever acts as a contact profilometer, which generates a pattern of the sample surface upon the displacement of the tip relative to the roughness on the surface. As the AFM tip maps the sample surface, the cantilever bends owing to the repulsive forces between the samples’ surface and the tip. The cantilever also deflects due to the pressure applied to the tip and the sample creating a topography of the sample surface as a feedback loop.

The AFM-Raman technique has recently emerged as an advanced tool to image the specimen topography and roughness at a nanoscale through AFM as well as chemical composition and functional groups through Raman spectroscopy. Tip-enhanced Raman scattering (TERS) uses local surface plasmon resonance effects at the probe tip to acquire physicochemical information including covalent bonding via non-metric spatial resolution. TERS is found to be promising for characterizing 1D and 2D nanomaterials, semiconductors, organic compounds and polymers.

### 2.1. AFM Scanning/Operation Modes

**Figure 1:**
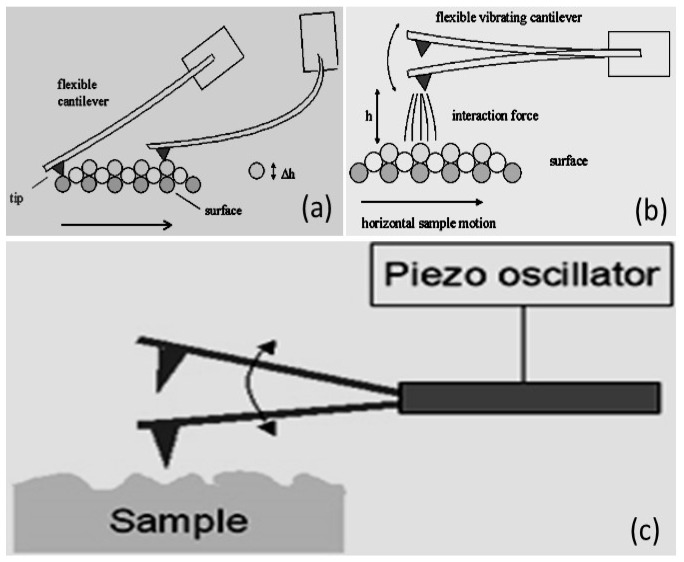
Different modes of scanning in an AFM: (**a**) contact mode; (**b**) non-contact mode, and (**c**) tapping mode. *The figure is designed by the authors*.

AFM can be operated under various modes, while the choice of suitable operating mode depends on the desired information and types of AFM image. In summary, there are three main operation modes of AFM, such as tapping mode, contact mode and non-contact mode. In addition to their static or dynamic nature, the three modes depend on the proximity and interactions between the sample surface and the tip (Figure 1) [81].

#### 2.1.1. Contact Mode

Contact is mostly named a static mode, while some researchers prefer to designate the operating modes through their detection mechanisms [82]. It is characterized by a permanent repulsive interaction between the tip and the sample. The tip ‘follows’ the relief of the surface in the manner of a feeler device. The main advantages of this operation mode are the speed, the simplicity of implementation, and the possibility of simultaneously measuring another parameter, i.e., mechanical (friction) or electrical (resistance). Its notable drawbacks are the possible damage to fragile samples, the rapid wear of certain tip coatings, as well as the inconvenience linked to the presence of contamination on the surfaces [83].

#### 2.1.2. Non-Contact Mode

In this operating mode, the cantilever vibrates at its natural resonant frequency and low amplitude away from the sample surface. Approaching it, the attractive and repulsive forces modify the resonance frequency of the lever coupled to the surface. Controlling this frequency makes it possible to regulate the tip and sample distance. It then becomes possible to map the surface of the sample.

For non-contact operation mode, a vacuum is not always required compared with scanning electron microscopy (SEM) or transmission electron microscopy (TEM) and the samples can be scanned in a liquid medium or air. These advantages have made AFM ideal for research biomaterials in the native or quasi-native environment as well as in real-time and samples can be pictured in liquid or surface air. The major advantage of this technique is that it does not cause any tip wear or damage to the sample. Its main drawback is poorer spatial resolution. It should be noted that by working with conductive or magnetic tips, it is possible to be sensitive to variations in electrostatic or magnetic forces (e.g., electrostatic force microscopy (EFM) and magnetic force microscopy (MFM)). Having experience with this mode indicated that soft materials can be easily damaged due to the shearing deformation and/or large normal force. Also, a sharp tip apex may destroy due to the imaging of a rigid sample in contact mode [84].

#### 2.1.3. Tapping Mode

This mode was developed to improve the resolution compared to the non-contact mode. For this, the operating principle remains the same, but with greater amplitude of oscillation so that at each cycle the tip crosses the attractive area until it strikes the sample at the edge of the repellant area. At the usual frequencies of this technique (100–300 kHz), it is only in contact with the surface over very short time intervals, thus making it possible to minimize the effect of lateral forces compared to the contact mode. Soft and/or fragile samples are, therefore, not damaged, and objects weakly anchored to the surface of the sample do not move. Moreover, the tip wears less quickly. In practice, the frequency is fixed, close to the resonant frequency, away from the sample. Approaching it, the attractive and repulsive forces then modify the range of oscillation of the lever. Controlling this amplitude makes it possible to regulate the tip/sample distance and to constitute a topographic image. The main advantage of this intermittent mode is the virtual absence of lateral forces, and therefore very little wear on the tips and a greater diversity of measurable samples. Its major drawback is that the force exerted, admittedly reduced compared to the contact mode, is difficult to control.

### 2.2. Phase Imaging

**Figure 2:**
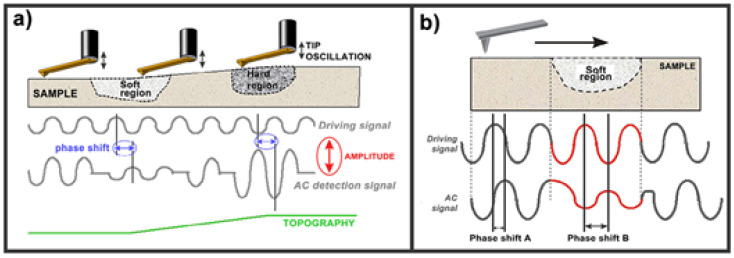
Lag changes due to the difference of mechanical properties of sample surface: (**a**) mode of modulation and (**b**) typical force curves [33,85]. *Reproduction with permission of Elsevier*.

Phase imaging allows mapping variations in surface properties of polymer materials due to the difference in terms of adhesion, friction and elasticity forces. This results in phase lag monitoring between the output signal and the cantilever oscillation (Figure 2). Changes in the phase lag result in a change in the physical properties on the sample surface [85]. Phase imaging, a method closely connected with IC-AFM, enables surface properties to be observed beyond pure topography. This results in a phase change as well as the lower amplitude of the cantilever oscillation. Since this phase change is a feature of the sample material’s energy absorbency, it is characteristic for rigid or soft materials or, more generally, low and high-energy absorbency materials (Figure 2) [86].

Phase imaging enables the study of the dissemination of various materials or processes in the sample, e.g., phase differentiation among lipids [87] or drug delivery inside nanoparticles [88].

### 2.3. Atomic Force Microscopy

AFM can be used to determine the variation of the pressures between the AFM tip and the surface sample, thereby producing curves of force-distance [89]. A sort of forces can be spotted. These consist of van der Waals forces, Coulomb forces, electrostatic forces, or forces in ligand-receptor pairs [90,91,92,93,94]. As mentioned above, the principle of AFM is based on determining the forces between the tip of the AFM and the surface of the sample. Atomic Force Spectroscopy (AFS) has several advantages over other techniques such as: (i) the stylus profile meter that is the probe’s high lateral positioning, enabling the sample’s behavior to be observed at the nano-scale resolution, and (ii) high-sensitivity force, enabling forces to be recorded down to the PN range [95].

### 2.4. Cantilever-Tip Systems

One of the furthermost essential tools to obtain images at the nanoscale is AFM that applies a cantilever with a sharp probe scanning on the sample surface. Hooke’s law helps better understand the forces between the AFM tip and the sample surface where a laser beam senses the deflection. The cantilever can be supposed as a spring. The force between the sample surface and the AFM tip is related to the cantilever’s spring constant (i.e., stiffness) as well as the distance between the tip and the surface [96].

Hooke’s law [96]:

(2) F=−k·x

where F = force, k = spring constant, and x = cantilever deflection.

For high-resolution imaging by an AFM tip, its structure, radius and chemical composition are the key factors. The key limiting factor concerning an AFM’s overall resolution is the tip apex radius. Highlights in the tip’s size range are distortedly and extended, smaller highlights may not be apparent at all [97]. As the cantilever’s spring constant is less than air, a folding of the cantilever happens, and this deflection is tracked [98].

The nanoprobes are often used as a metal-coated tip to have additional properties, such as conductivity, in scanning probe microscopes. However, this method is more costly because the specimens can be harmed by the diamond coatings [98]. New studies have developed prototypes of nanoprobes that are coated with different materials. For instance, Dai et al. [99] have developed a conventional scanning probe microscope (SPM) probe tips by adding multi-walled carbon nanotubes (with 1 μm and 5–20 nm diameter) to their apex using manual manipulation and optical microscope epoxy [100]. Many works [101,102,103,104,105,106] have shown that graphene can be a suitable coating material to extend the lifespan of a conductive nanoprobe. This prototype offers better mechanical durability with high conductivity without raising the tip radius and/or changing the spring constant of the cantilever. Its mass is minuscule compared to hard coatings like diamond that have a substantial mass. Graphene may also be used to functionalize the probe’s surface with piezoelectricity and hydrophobicity properties [100].

The cantilever and the tip placed at its end are very important elements. Derived from complex microfabrication processes, there are different types, different shapes and composed of different materials. We will describe in this paragraph the type of lever/tip systems that we mainly used for our work. These are cantilevers formed of a rectangular silicon beam with an aluminum coating, on the upper part, to better reflect the laser spot. Depending on the dimensions of the lever, different stiffness values are available, modifying the pressing force exerted for a given deflection. We used levers with stiffnesses between 0.2 N/m and 40 N/m. The points integrated into these levers are of tetrahedral geometry

**Figure 3:**
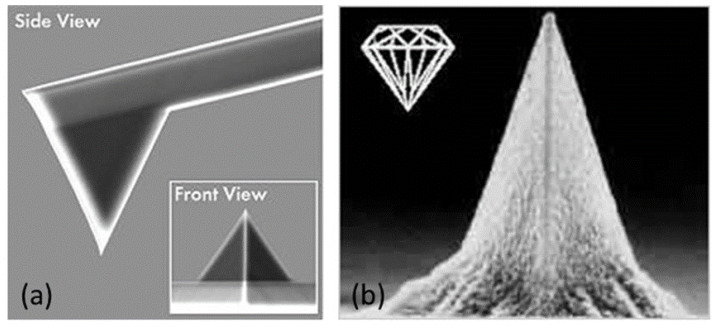
(**a**) AFM Si_3_N_4_ tip with Cantilever and (**b**) diamond coated AFM tip, adapted from [109]. *Reproduction with permission of Nanomonde Co.* (*USA*).

Microfabrication etching technique is one of the most commonly used methods for preparing AFM tips. The cantilever/sample systems are etched using this method from oxidized silicon wafers using photographic masks to describe the cantilever’s form. This leads to tips with a radius below 30 nm. Super tips can be generated at the end of pyramidal Si_3_N_4_ tips through the regulated growth of carbon filaments (Figure 3) [107]. The tips developed by these techniques are later modified by surface yielding well-defined sensor systems at the nano-resolution to characterize the physicochemical, mechanical, and elastic properties of the sample. For example, nano-sensors can be produced by the covalent binding of DNA molecules based on a wide range of chemical reactions [108].

### 2.5. Principle of AFM-IR Technique

**Figure 4:**
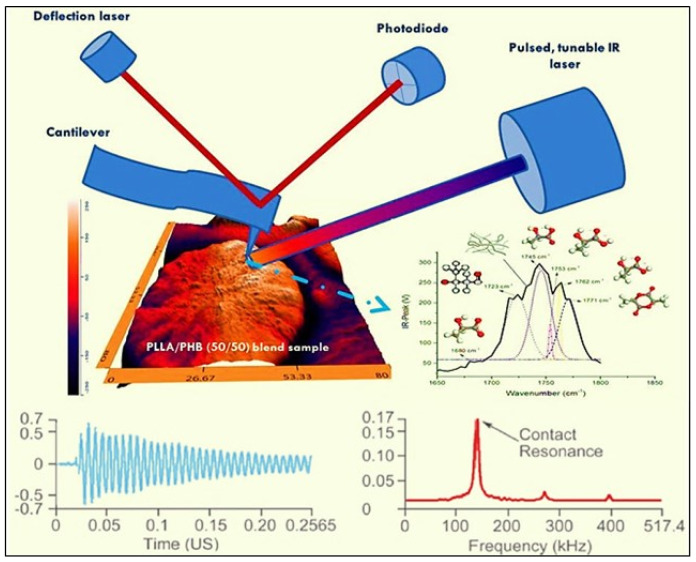
Principle of AFM-IR technique. Insert image shows an example of carbonyl groups of the blends of polyhydroxy butyrate and polylactide acid (50/50). *This figure is designed by the authors*.

AFM-IR is a combination of an AFM and a tunable IR radiation to detect a photo-thermal effect and access chemical information down to a nanoscale resolution [110]. Through AFM-IR, the limit resolution of sub-diffraction is obtained. This is done via tracking the diversion of an AFM probe which is in contact with the sample surface and is deflected by rapid transient thermal expansion of the sample following of the absorbance of an infrared pulse. This has already been seen to associate well with measured infrared absorbance using traditional macroscopic FT-IR [111]. The principle of AFM-IR [112] is to couple the AFM in contact mode (or tapping mode) with a pulsed tunable laser is shown in Figure 4. In the first-generation AFM-IR, a sample is irradiated near field infrared radiations or laser through an infrared-transparent prism (ZnSe). The photo-thermal temperature of the sample increases when the wavelength of the laser diode is within the sample’s absorption bands. The local rapid thermal expansion of the sample takes place as it heats up by absorbing radiation from the laser. The AFM cantilever senses this rapid thermal expansion of the sample through pulses that excite resonant oscillations. However, ring-down mechanisms can gradually decay these oscillations (Figure 4).

Special data acquisition electronic systems measure the cantilever deflection concerning the laser pulses. By extracting the frequencies of the oscillations and amplitudes by Fourier techniques, the ring-down mechanisms can be studied. Local absorption FT-IR spectra, similar to standard transmission spectra, can result from the measurement of cantilever oscillations, which is dependent on the source wavelength. Besides, the oscillation frequencies of the ring-down determined using the Fourier techniques can also be used to qualitatively measure the sample’s mechanical stiffness. The single wavelength of the IR source can determine the sample’s mechanical properties and surface topography. The new AFM-IR system (nanoIR2) can also perform local thermal measurements of the surface of a sample. These measurements are done by bringing a probe capable of being heated in contact with the sample’s surface. The probe is typically a microfabricated structure made of silicon with a sharp tip at the end of a long cantilever extending from a substrate. The sharp tip contacts the sample surface while the long cantilever allows sensitive force control between the tip and sample. The probe is resistively heated by flowing current through a conductive path in the cantilever. The system will automatically ramp the probe temperature and record the deflection of the probe along with several other possible signals. The probe can be manually positioned at many locations or the system also has the capability using the Array function to measure the sample over an area on the sample’s surface.

While in the top-down method, the advantages of scanning probe lithography are high-resolution molecular, chemical, and mechanical non-patterning capabilities, accurately controlled non-patterns are limited for high throughput applications and manufacturing. Although, the AFM allows analyzing samples with a spatial resolution order of 50–100 nm. In some cases, it will be impossible to prepare the sample with such a thin film (under 1 µm). For example, in the metal sector or silicon materials, it is very hard to prepare a thin film under 1 µm by using a microtome. To overcome this drawback, Baden et al. [113] adopted a new approach by using the focused ion beams (FIB) instead of the preparation of thin-film by a microtome. Although this method may lead to several deteriorations of the substrates due to the ion bombardment attacks and local thermal heating. However, the damages on the studied polymer surface (polyimide) by ion beam irradiation were negligible and the chemical structure of polyimide before and after treatment with FIB remains mostly unchanged. This kind of information is very interesting because it helps to overcome the drawbacks of this new technique at least with polyimide. The analysis of ultrafine particles (UFPs) needs higher sensibility equipment. For this purpose, a new resonance-enhanced method to increase the sensibility of AFM-IR by using the Lorentz contact resonance (LCR) imaging mode is proposed.

With this improvement, it is possible to perform nanoscale IR spectroscopy on extremely thin films to even 5 nm thick [55]. In other words, with the new devices, AFM-IR (models: NanoIR2s, NanoIR3, commercially available from Anasys Instruments Inc.) can be able to investigate single polymer lamellae, self-assembled monolayers and biological membranes. The responsivity of AFM-IR can be also improved by using a quantum cascade laser (QCL) [54,114]. To demonstrate a spatial resolution higher than 50 nm, it is necessary to tune the repetition rate of the QCL that can match AFM cantilever’s one of the contact mode resonance frequencies. A spatial resolution of 25 nm has been reported to be achieved along with the sensitivity of self-assembled monolayers by using top-down illumination employing a gold-coated substrate and gold-coated AFM tip. The synchrotron IR beam in the resonance-enhanced AFM-IR configuration is amplitude modulated using a mechanical chopper. The mechanical chopper should be accustomed to a cantilever contact-resonance frequency. However, it should be noted that the interactions between the AFM tip and the sample surface determine the cantilever contact-resonance frequency. Noisy signals or complete loss of signals can emerge in the measurement if the resonance conditions are not properly maintained. Failure to correctly manage this resonance state may make larger noise levels in the calculation and/or lead to the total loss of signal [115].

Another mode is also used with AFM-IR, so-called the scanning thermal microscopy (SThM) mode. This is a mode where a special AFM tip scans the surface of the sample surface via the contact mode. This tip has a resistive element near its apex. As the tip changes temperature due to variations in the thermal conductivity or temperature of the sample in contact with the tip, the resistance of this element changes. This resistance change is monitored by the hardware using a Wheatstone bridge circuit and can be output to the controller to generate an image. To run experiments, you mount the sample onto a substrate, place it on the sample stage, and obtain an image of the sample’s surface in the contact mode, following the normal AFM procedure but using a resistive thermal probe. Later, the user applies a voltage to the resistive thermal probe, balances the Wheatstone bridge and measures a voltage which is characteristic of the resistance of the probe. This voltage displayed along with the other images in the Analysis Studio software.

Surfaces of samples having sharp curvatures can concentrate the electromagnetic fields via the “lightning-rod effect” [116]. Such an effect improves the field confinement due to the sample surfaces sharp edges, which can provide increased sensitivity [117]. Recently, Dazzi et al. reported the AFM-IR using tapping mode, which complemented the AFM-IR’s contact mode. This technique can be appropriate for samples with soft nature or loosely packed surfaces including polymeric nanoparticles [118,119,120]. In the tapping mode, the cantilever oscillates vertically, near the resonance, which can reduce or eliminate the shear forces posing the risk of damage to the sample surface or the tip during scanning [121]. A spatial resolution of 10 nm for soft samples can be attained using the tapping AFM-IR technique with a heterodyne detection scheme [118,119].

### 2.6. Nano-Thermal Analysis (Nano-TA)

**Figure 5:**
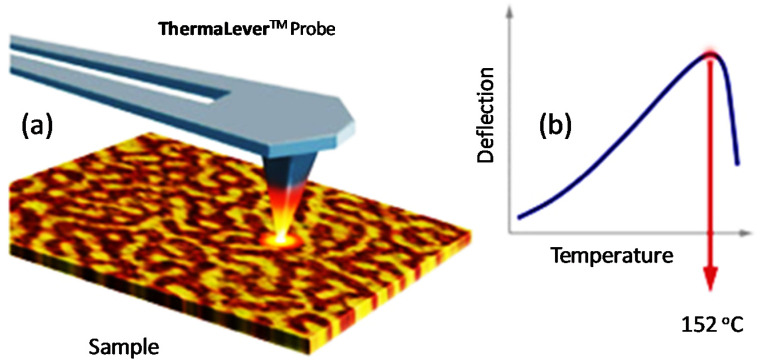
(**a**) Nano-TA uses a heated AFM tip to measure (**b**) thermal transition of polymer. *Reproduction with permission of Brucker Inc.* [123].

The other significant feature of AFM is about its attitude to be coupled with thermal analysis to better characterize the thermal properties of heterogeneous samples in different regions. Also recognized as nano-thermal analysis (nano-TA), this groundbreaking AFM-related method enables a local thermometric analysis [122]. This technique is often used to measure local thermal transitions of materials that can be imaged with AFM. A special thermal probe with capabilities to be heated under controlled conditions via the applied voltage is used for this analysis (Figure 5). The probe is subsequently relocated to the point of interest while increasing its temperature at the same time after imaging the sample surface. The temperature from the probe tends to locally heat the sample coming in contact with it. During this thermal ramping process, any deflection or resistance by the probe is recorded along with the power applied. Figure 5 illustrates the probe’s initial upward deflection caused by the sample’s thermal expansion followed by its penetration into the sample from the localized softening or melting of the sample’s surface. This phenomenon occurs at the glass transition temperature or melting temperature of the sample due to a high resistance region of the probe (e.g., standard silicon probe).

There are two styles of micromachined ThermaLever probes available. One has a cantilever length of ~300 μm and the other has a length of ~200 μm. The ThermaLever probes have tip heights of 3–5 μm and a large area to reflect the AFM laser. The 300 μm cantilevers can achieve a maximum temperature typically of 400 °C and the 200 μm cantilevers achieve a maximum temperature of 350 °C. Both styles of probes are made from doped Silicon which means that the temperature change with resistance is nonlinear. This means that when calibrating the probe temperature, three calibration samples are required to accurately calibrate the probe. Also, the probe will have a maximum temperature as mentioned above. This maximum temperature is the highest temperature that can be achieved before the resistance turnaround point.

The main advantage of testing nano-scale thermal analyzes is the detection of changes in the polymer crystal state, particularly the glass transition temperature (T_g_) and melting temperature (T_m_), without affecting its mechanical properties. During the measurements, the cantilever is heated while the probe is moved toward the specimen until its expansion due to the cantilever is local heating (Figure 5). The sample swelling pushes the probe up and causes a rise in the vertical bending of the cantilever. Therefore, this deflection is assessed by applying AFM photo-detector device based on an AFM standard. The specimen changes its internal temperature due to the change in transition temperature at the softer state than the initial state. Hence, the cantilever bending decreases and the sample is forced to induce elastic or plastic deformation [122].

## 3. Application of Advanced AFM in the Crystallization of Polymers

### 3.1. AFM in Polymer Crystallization

#### 3.1.1. Primary Stages of Crystallization

AFM is likely used to exclusively investigate the crystallization of polyolefin polymers at the nanoscale level [123,124,125], especially in the initial stages of crystallization. For example, the classical nucleation theory anticipated the development of ephemeral embryos in variable sizes. The embryos can grow, shrink, and probably stochastically disappear, but a Boltzmann distribution characterizes the overall population. This object only becomes a stable crystal nucleus as the critical size is attained by an embryo. At this stage, the transition-driving free energy offsets the surface energy [73]. In poly(bisphenol A octane ether) or PBA-C8 [126] and elastomeric PP, these germinations were recorded as transient dots [127]. Both the polymers gradually crystallize at room temperature, thus making in-situ observations easier.

**Figure 6:**
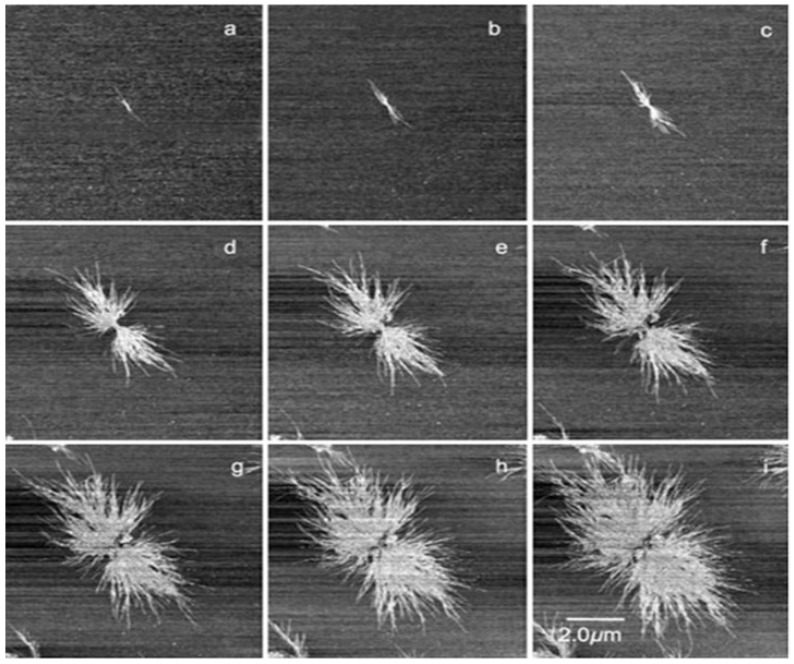
Phase images of spherulite development in PBA-C8 at different crystallization times: (**a**–**i**) The temperature was 30 °C and the overall time was 167 min. *Reproduction with permission of Elsevier* [126,128].

When a stable nucleus is shaped, it is possible to follow in-situ growing and splitting of a “founding lamella” seen edge-on. Figure 6 shows the primary stage of nucleation followed by the initial growth of an edge-on founding lamella of PBA-C8 and its subsequent branching [128]. The growth phase and branching of poly(ethylene oxide) or PEO have also been reported by Schönherr [129]. The primary nucleation or spherulite formation occurs either homogeneously (as described above) or heterogeneously (with single lamella grown in the melt or primary nucleation from a preceding surface) [130]. For instance, 6 edge-on lamellae developing from a shared center can be found in the case of PBA-C8 [126,128].

The typical mode of spherulite formation can be diagnosed mostly through single-angle splitting with the repetitive production of new lamella near and parallel to the newly formed lamella followed by their widening [131]. Although low-angle branching is an essential feature in the formation of spherulite, the dominant cause of such splitting is not universally agreed. Chan et al. [126] suggested that the non-crystallographic and low angle splitting of lamellar is caused by the induced nuclei. It is assumed that cilia or any unsecured folds on the basal surfaces of the parent lamella are organized as the embryonic crystals tied to the parent structure.

With a mean of roughly halfway between T_g_ and T_m_, the temperature required to induce nucleation and lamellar growth is almost equivalent (Figure 6). Firstly, an induced nucleus appears as stable bright spots contiguous to the primary lamellae (Figure 6a–c), which further develop into secondary lamellae (Figure 6d–i).

#### 3.1.2. Effects of Film Thickness

**Figure 7:**
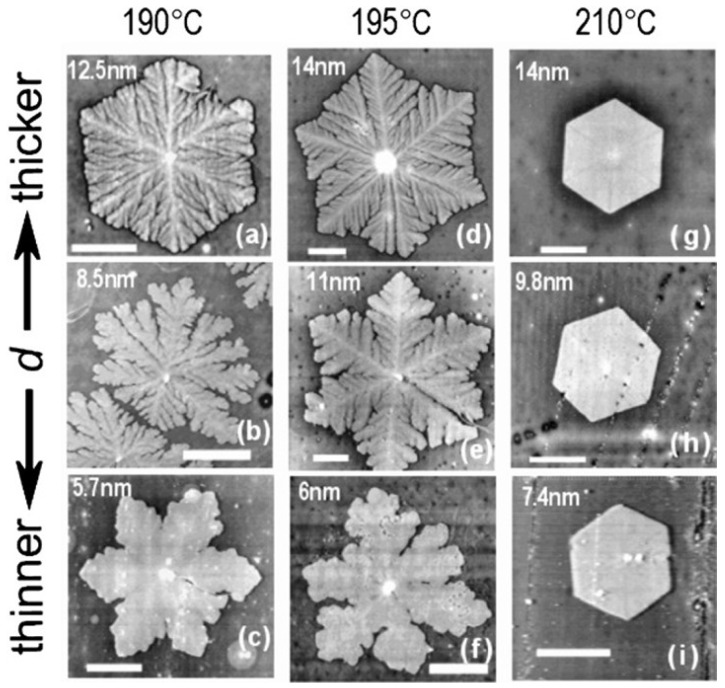
AFM images of crystals grown from ultrathin films of iPS at different temperatures and sample thicknesses. *Reproduction with copyright permission from Elsevier*, *Prud’homme R.E* [135].

Investigations on constrained systems phase transitions and polymer crystallization in thin and ultra-thin films have gained interest due to AFM’s popularity. Liu and Chen [132] wrote have extensively reviewed this subject, a few notable points are summarized here. Crystallization leads to edge-on oriented lamellae and growth rates are similar to those in bulk for molten thin films (thickness > 200 nm). For thin films with a thickness of 100–200 nm, a sudden transition is noticed from edge-on lamellae to face-on lamellae. The rate of growth of the well-studied isotactic polystyrene (iPS) system is G(d) = G(1-ad-1) where “G(1-ad-1)” is the bulk rate and the parameter “a = 6 nm” is insensitive to the crystallization temperature, substrate and molecular weight [133]. The reason for this slowdown in the ultra-thin film regime’s crystallization rate is not recognized. A stronger dependence of G(d) on d-1 for PEO grown on an attractive substratum has been reported. Once the thickness of the film exceeds the thickness of the lamellar lc ≈ 10 nm, extra changes are seen. The reduced (thinner) melting regions around each growing crystal are observed here. G(d) dependence on d-1 is reduced. As shown in Figure 7, the lamellae show that the growth morphology at any given temperature can be stiffer and flexible.

The diffusion length δ = DG^−1^ is a suitable parameter to realize some features of crystal morphology. In this case, D is the diffusivity [m^2^·s^−1^] of matter, mechanical, or thermal energy, which affects the crystallization. The extent of π is an indicator of the field immensity in front of the crystallization axis. It can be higher for slow growth rate undercooling (close to T_m_) and smaller for rapid growth rate at lower T_c_, where G is larger, and D is abridged. The rate of growth G is constant in time in all experiments considered here, maintaining consistency with the crystallization rate interface control. Furthermore, the morphology of the crystal can be influenced by the diffusion without imposing a dependency of t^−1/2^ on the growth rate (G). The development of a smooth topological interface is unstable to local contour variations. Mullins-Sekerka instability can be explained by the development of fast-growing fingers and associated thin characteristics with a transverse size proportional to (DG-1)^1/2^ [134].

With numerous potential advantages by combining a nanoscale morphological characterization and chemical composition identification, the AFM-IR is a worthwhile analytical tool for the chemical characterization of a variety of materials, especially for structure-property relationships. We describe here the latest progress in the application of this technique to identify, verify the miscibility, interaction, aging and crystallization of polymer, as well as related phenomena, which cannot be done with traditional AFM techniques [136]. Moreover, the information of these analyses is limited in the literature.

The direct visualization of primary nucleation at an early growth stage has been made possible through AFM imaging of polymer crystallization, which was previously unapproachable. The findings obtained through AFM have mostly complemented derivations from other methods or existing theory. However, several new questions have evolved, particularly concerning the understanding the branching caused by induced nucleation, operation of huge screw dislocations and the identification of polymer during crystallization.

### 3.2. AFM-IR in Polymers

#### 3.2.1. Polymers Blends

For blend materials, the understanding direct determination of the miscibility, understanding polymeric separation and chemical interactions in a formulation are crucial to controlling the final properties. However, it remains key challenges due to the lack of correlated imaging tools at the sub-nanometer and chemical characterization. The first application of AFM-IR in polymer blends that should be mentioned was the work of Prater et al. [137] in which they tried to use AFM-IR to characterize a tertiary blend of common commercial polymers including polycarbonate (PC), epoxy resin (ER), polystyrene (PS). The AFM-IR technique can provide infrared spectra series in the C–H stretching region at different positions on the sample surface. The amplitude of the cantilever oscillation is related to the amount of infrared radiation absorbed by the sample [111,132,133,134,135,136,137,138,139,140,141,142].

The obtained results showed changes in terms of peak intensities of the C–H bonding in the spectral range of 2920–2970 cm^−1^ as the tip moved from PC to PS domains. Polycarbonate (PC)/Acrylonitrile butadiene styrene (ABS) blends are one of the most popular materials used in automotive and electronics devices. This is due to their excellent properties such as toughness, resistance to heat and ability for high flow during molding. AFM-IR can be used to explore the deflection of PC/ABS blends by setting the IR source to a constant wavenumber before scanning. The characterization and distribution of polymer in ABS/PC composite were performed by recording the nano-IR spectra in the C–H stretching region of 2800–3200 cm^−1^. The aromatic C–H stretching vibration in polystyrene (PS) was found at the IR band of 3028 cm^−1^. The CH_3_ stretching band at 2960 cm^−1^ assigned to PC and CH_2_-stretching band at 2932 cm^−1^ attributed to polystyrene (PS) exhibit significant changes when the AFM cantilever scanned the sample surface to provide the chemical composition of ABS/PC at the nanoscale [143].

Felts et al. [140,144] have investigated several polymer systems, including polyethylene (PE), polystyrene (PS), and poly(3-dodecylthiophene-2,5-diyl) (PDDT), for their chemical characterization, nanoscale imaging and tip-based nanofabrication with spatial resolutions over 100 nm. The polymer nanostructures were written near a similar substrate, with some nanostructures overlapping. The position of absorption peaks at 2926 cm^−1^ was attributed to asymmetric C–H stretch. The band at 2860 cm^−1^ was assigned to symmetric C–H stretching. These peaks can shift to lower or the higher frequency with a variety of feature sizes. This is because of the thermal expansion of polymer blends, which depends on the feature size. Consequently, the cantilever response amplitude can lower with a decrease in the polymer feature size. In the case of PS and PDDT, although AFM-IR spectra show a low signal-to-noise, due to artifacts of laser power drift, the authors can still distinguish between nanostructures of polymers [145].

The determination of the miscibility of amorphous components in polymer blends used in pharmaceutical systems was also investigated by AFM-IR. Van Eerdenbrugh et al. [59,146] used AFM in combination with mid-IR spectroscopy to study the miscibility of an equimolar binary blend between poly-(vinylpyrrolidone) (PVP) with dextran or maltodextrin (DEX). By analyzing infrared absorption peaks at 1280 and 1350 cm^−1^, the rich-DEX or rich-PVP domains were distinguished. More specifically, for pure DEX with a vibrational mode of (CH_2_ wagging; CH_2_ twisting) [147] and, the intensity of the band at 1350 cm^−1^ was higher than that of the 1280 cm^−1^ band, while for PVP with vibrational modes of C−H bend; CH_2_ wagging, C−N stretch [148], the opposite tendency was observed. The AFM-IR results showed that the large discrete domains correspond to DEX-rich phases, while PVP enriches the continuous phase. The chemical and topographical images were in accordance.

In the petroleum and natural gas industry, the use of hydraulic fracturing techniques to crack rock is one of the most popular methods to release crude oil or natural gas by using sealing materials that can withstand ultra-high temperatures and high pressure (Ultra HTHP) [149]. These materials are often fabricated by a blend of high-performance materials including polytetraflouroethylenes (PTFE), polyaryletherketones (PEEK), perfluoro-elastomers (FFKMs), and ethylene–propylene diene monomer (EPDM). A good understanding of the compatibly, the micro, nanostructure and the chemical properties of the blend is the key factor to improve their design and their process development. AFM-IR spectra helped chemical identification of each pure polymer component in the blend and thus can help to optimize the blending conditions [149]. AFM-IR spectroscopy and imaging can provide information for controlling the convergence of processing conditions to obtain a desirable mixing, which is ideal for a better performance of the end-use polymer [82].

Purohit et al. [58] investigated the miscibility of the drug-polymer amorphous solid dispersion (ASDs) of Itraconazole (ITZ) and hydroxypropylmethylcellulose (HPMC) blend through AFM-IR spectroscopy and fluorescence imaging. Both these techniques provided valuable information about ASDs microstructures. The ASD components underwent mixing upon the heating of the films. The authors affirmed that the AFM-IR is useful to characterize the microstructures in a drug-polymer system as well as their miscibility.

AFM-IR technique can help interpret the phase behavior of polymer blends that could be partially miscible such as poly(vinylpyrrolidone) (PVPK90) and hydroxypropyl methylcellulose acetate succinate (HPMCAS) to fabricate nanofibers for a tunable drug delivery system and nanoscale phase separation in electrospun blend fibers [150].

Recently, the miscibility of a polymer blend based on a pharmaceutical drug Telaprevir with three different polymers, an amorphous solid dispersion drug, was evaluated using nanoscale-IR spectroscopy, thermal analysis, and Lorentz contact resonance measurements [151]. The study indicated that AFM-IR is useful for phase separation and characterization of the microstructure of solid dispersion with respect to the polymer nature. According to the AFM-IR results, the continuous phase was enriched with polymer while the drug-rich phase formed discrete domains. These domains varied in size (from 50 nm to a few hundred) as a function of the system [151].

**Figure 8:**
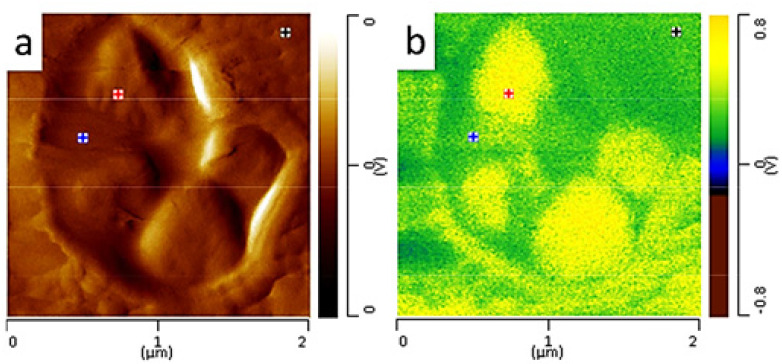
(**a**) AFM image and (**b**) AFM-IR image at the 1456 cm^−1^ showing the presence of polypropylene (PP) in the rubber nodules in a commercial high-impact polypropylene. *Reproduction with copyright permission from American Chemical Society*, *Tang* et al. [57].

Tang et al. [57] used the AFM-IR technique on a commercial high-impact polypropylene to study the composition of its nano-domains. These consisted of mainly polypropylene along with some polyethylene (PE) and ethylene-propylene rubber (EPR) [57]. These materials possess multi-level phase structures and contain rubber particles in their core-shell with the dispersion of the internal structures in a continuous PP matrix with the order of several hundred nanometers as shown by AFM mapping images (Figure 8). The chemical characterization of this core-shell is impossible with a traditional FT-IR. Polypropylene (PP) exhibits a strong peak at about 1454–1457 cm^−1^ which is assigned to the vibration of a methylene group (–CH_3_) in the PP side chains while both polyethylene (PE) and PP show a strong peak at the 1378 cm^−1^ band due to the vibration of symmetric bending of –CH in the main chains. The AFM-IR spectra image at the 1456 cm^−1^ band (Figure 8b) helps to confirm the variation of PP concentration in such blend. In other words, by using the AFM-IR, they confirmed that the rigid cores consisted of mainly rubber particles of PP, not PE as previously described before. More recently, they published also a similar work on the use of both nano-TA and AFM-IR techniques to evaluate the chemical composition and structure of a high impact polypropylene (HIPP) [152]. Also, the principal component analysis (PCA) is one of the most significant and powerful techniques in chemometrics, besides in a wealth of other areas [153]. PCA is a method for decreasing the dimensionality of such datasets, increasing interpretability while minimizing information loss [154].

#### 3.2.2. Crystallization of Miscible Blends

**Figure 9:**
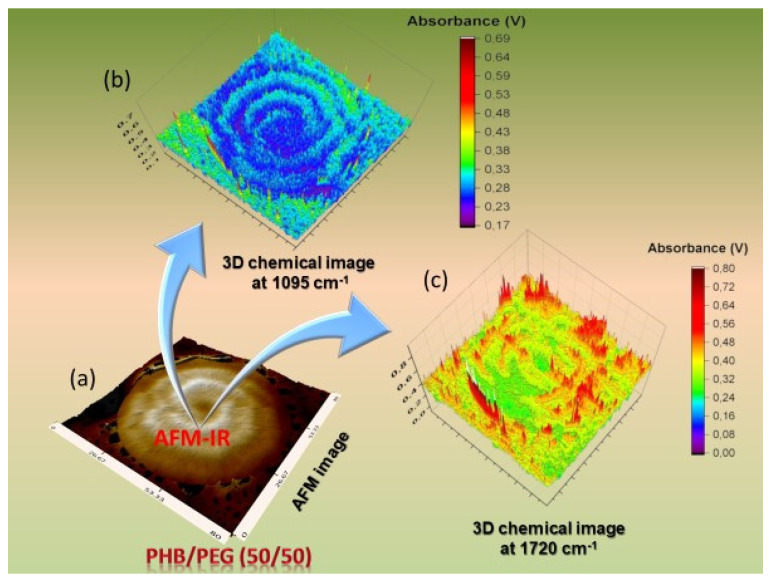
The PHB/PEG (50/50) blend isothermally crystallized at 40 °C: (**a**) tridimensional chemical image at the 1720 cm^−1^ band; (**b**) 3D AFM image and (**c**) 3D chemical image at the 1095 cm^−1^ band. *Reproduced with copyright permission from American Chemical Society*, *Nguyen-Tri* et al. [159].

The investigation on the distribution of polymer in miscible blends, especially on the sub-micrometer level is a practically difficult assignment despite the current advances in numerous X-ray techniques and microscopy [155,156,157,158]. AFM-IR has been used to examine the behavior of crystallization and diffusion in crystalline blends of poly(3-hydroxybutyrate) (PHB) and polyethylene glycol (PEG). The addition of PEG helps to improve the biodegradation of PHB and extends its applications. However, the presence of PEG will perturb the crystallization of the PHB. By using the AFM-IR, it was possible to detect and derive a 3D polymer diffusion in this miscible blend by following the variation of the bands at 1095 cm^−1^ (C–OH stretching) observed in the PEG and 1720 cm^−1^ (C=O) found in PHB (Figure 9) [159].

#### 3.2.3. AFM-IR in the Crystallization of Immiscible Blend

There are some works reported in the literature on the use of AFM-IR [160,161,162,163] to examine the behavior of polymer crystallization and diffusion in complex systems including equimolar miscible and immiscible crystalline blends at the nanoscale level [159,160,163]. Such studies used AFM-IR to analyze the crystallization behavior of an immiscible biopolyester based on polycaprolactone (PCL) and polyethylene glycol (PEG). The crystallization of this system is complex because they have a very similar melting temperature T_m_ and, thus, is expected to have simultaneous crystallization. However, the polymer phase separation at the nanoscale and the distribution of polymer in the sub-micrometer structure are limited. Thus, the mechanism of the crystallization process remains unexplored. The crystallization of an equimolar blend is first carried out at different temperatures between 30 and 40 °C near the melting temperature and followed the technic of polarized optical microscopy (POM).

This demonstrates that, when the crystallization temperature goes lower than 37.5 °C, the crystallization has undergone in two steps. The first polymer crystallizes first to form spherulites and then suddenly, the nucleus of another polymer appears in the boundary of spherulites of the first polymer and grows perpendicularly to the interface. The crystals of the second polymer cannot develop to the spherulitic structure. When the temperature is higher than 37.5 °C, one step crystallization is observed. The transition from two steps to one step crystallization is perceived at 37.5 °C where both inside and outside rejection are found. For the identification of the nature of each polymer in the blend, the AFM-IR spectrum of each pure component is first measured to determine the characteristic peak of each polymer. In this case, PCL exhibits a strong peak at about 1725–1728 cm^−1^ due to the presence of the carbonyl group, while this peak is absent in the PEG [160].

When the blend is quenched from the melt to an isothermal crystallization temperature of 40 °C, POM images show one-step crystallization of blend and the core-shell blended spherulites are not observed in this condition. Information about phase separation and the growth of PEG crystals are ignored due to the resolution limits of POM. In several blends systems with a large difference of melting, POM equipped a hot stage that can be used to determine the identification and the distribution of polymer blend by heating overcome the melting of a polymer but lower than that of the other. The lower melting point polymer will be melted first and becomes darker under polarized optical microscopy observation. However, this destructive method is limited with blends having similar melting points like PEG and PCL.

**Figure 10:**
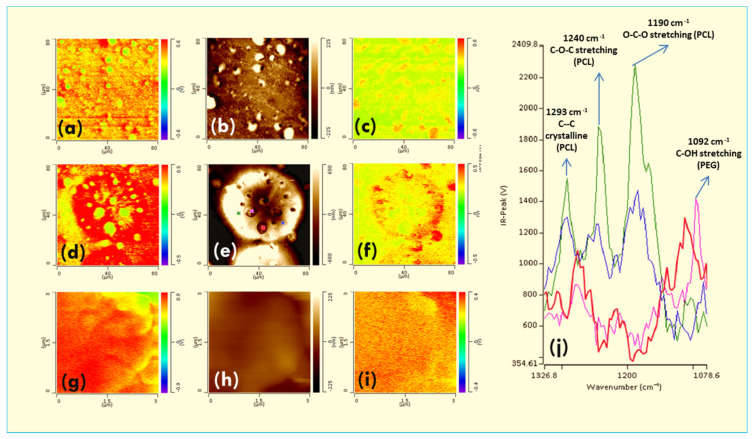
AFM images (**b**,**e**,**h**), IR mapping images (**a**,**d**,**g**) at 1724 cm^−1^ (carbonyl group of PCL), IR mapping images (**c**,**f**,**i**) and AFM-IR spectra (**j**) of PCL/PEG (50/50) blends isothermal crystallization at 40 °C. *Unpublished work*.

AFM-IR is used to better understand the crystallization process of the PCL/PEG blends. High-resolution AFM images show a liquid-liquid phase separation without crystallization (Figure 10b). At the beginning of the crystallization, free energy from the supercoiling process is not enough to cross over the energy barrier to form the stable nuclei and thus spinodal decomposition takes advantage of priority. With the crystallization time, in the PCL-rich domains stars to develop to nuclei and gathered together into spherulite-like features (Figure 10b). This structure continues to develop and occupy the entire sample surface including PEG rich regions. PEG-rich regions were blocked in the pre-existing PCL spherulites. The PEG rich areas nucleated and developed within PCL spherulite in confinement conditions. In this condition, the lamella density of PEG is lower than that of PCL and, thus, PEG crystals remain in the holes in the PCL template.

AFM-IR absorption maps of the carbonyl group of PCL at 1720 cm^−1^ can be used to examine approve these hypothesizes (Figure 10a,d). Figure 10a,d show a low IR absorption at 1724 cm^−1^ of small confined spherulites suggesting their main composition by PEG-rich domains. In contrast, IR absorption maps at 1092 cm^−1^ corresponding to the C–OH stretching in PEG represents a strong absorption in nodule structures. This allows confirming the PCL-rich domain belongs to bigger spherulites (c, f). The interface between two types of spherulites is also investigated by zooming in the small spherulite boundary at the sub-micrometers scale. Figure 10g–i represent the IR absorption AFM image map of the carbonyl group and IR absorption map of the C–OH group respectively. It can clearly distinguish two different regions containing PEG rich domains and PCL rich domains which are separated by spherulites boundary. It is very interesting to see that, in the PEC rich domains, there is also the presence of PEG lamella with the dimension of several tens of nanometers. This information is very interesting because, although because the blends are immiscible but the phase separation process during the cooling is not complete, the PEG is expected to be segregated into inter-lamella of PCL.

As acknowledged by the authors, for the first time, the distribution (or diffusion) of polymer in the blend can be directly observed at the nanoscale on a high-resolution AFM image. By using small-angle X-ray scattering (SAXS) and wide-angle X-ray scattering (WAXS) patterns, it is possible to speculate on lamellar structures in terms of their dimension, thickness and segregated mechanism. However, these analyses do not provide information on the morphology and direct observation of the lamella distribution in the blend at the nanoscale level. Full IR absorption spectra are also collected in PCL-rich domains (green and blue lines) and PEG rich domains (red and rose lines). It demonstrates that IR spectra are different from In the PCL rich regions and PEG rich domains. In the PEG-rich domain, there is a strong IR absorption band at 1092 cm^−1^ due to the C–OH stretching. However, the IR absorption intensity of this peak is weaker in the PCL region. In contrast, the IR absorption peaks at 1293 cm^−1^, 1240 cm^−1^, and 1190 cm^−1^ are attributed to the C–C stretching, C–O–C stretching and O–C–O stretching, respectively in the PCL regions.

### 3.3. Other Applications of AFM-IR in Polymer Science

With numerous advantages of combining a nanoscale morphological characterization with chemical composition identification, the AFM-IR technique is found to be quite useful for a variety of materials. Some of the latest progress in the application of this technique to identify, verify the miscibility, interaction and aging, as well as related phenomena, which cannot be done with traditional AFM, are described here [55].

For composite materials, the chemical characterization in the interfacial region between polymer matrix and reinforcements is very important to better understand the final properties and the failure mechanisms during the service lifetime of polymeric materials. The latter may help formulations to converge more efficiently on optimal processing conditions. AFM-IR has shown to be a useful tool for this kind of analysis.

#### 3.3.1. AFM-IR in Studying Polymer Aging

During the service lifetime the performance of polymers can deteriorate through the aging process due to environmental factors such as temperature, UV radiation and humidity [163,164,165,166]. This alteration can lead to the decommissioning of these products [167,168,169,170,171]. The aging of polymer material is manifested in physical and chemical degradations, a slow and irreversible process. The effects of this degradation resulting from the notion of the lifetime of the material, i.e., the time required a property (i.e., physical, chemical, or electrical) to reach a threshold below which the material becomes unusable. The term physical aging encompasses all processes leading to irreversible alteration of the material properties without a chemical modification of the structure of the macromolecules constituting the material. Physical aging [172] may result from the following: (i) changes in the spatial configuration of macromolecules (crystallization)-surface phenomenon; (ii) surface phenomenon (cracking of the surfactant environment); (iii) transport phenomenon (solvent penetration, migration of adjuvants).

Chemical aging [173] may relate from: (i) changes in the spatial configuration of macromolecules (crystallization)-surface phenomenon; (ii) surface phenomenon (cracking of the surfactant environment); and (iii) transport phenomenon (solvent penetration, migration of adjuvants). The main general types of reactions involved in chemical aging are the following: scission of polymer chains, depolymerization, crosslinking, and oxidation. Understanding the aging mechanism is crucial because it can provide information on the prediction of the service lifetime of polymer materials. This can help to avoid accidents due to the use of unsuitable polymers and to interfere with the aging process to accelerate or slow down the degradation. In this direction, investigations on the aging and stability of polymers are extensively realized. The recent advances in nanoscale analysis have shed light on aging investigations. AFM-IR technique has also been used to better understand the aging mechanism of common polymers and composites.

Morsch’s group has conducted several works on the use of AFM-IR technique to examine the aging processes of several coatings and polymers [174,175,176,177,178]. AFM-IR analyzed the chemical composition of aging products on the surface of a composite based on epoxy resin and silicon rubber. They used the electrical discharge to attack the composite surface and then investigate its effect on the chemical composition of the composite in the surface by both ATR-IR and AFM-IR. In many cases, ATR-IR gives similar results in aged and non-aged samples, meaning that the aging process does not affect the chemical structure of the composites. However, AFM-IR can provide more resolved spectra and nanoscale chemical mapping images of oxidative products (interfacial tracks and buried channels). The AFM-IR helps to confirm that the silicon rubber remains stable under these electrical attacks.

In another publication, both ATR-IR and AFM-IR techniques were used to investigate the degradation of oil paint used in the Picasso and Mondrian paintings [177]. The ancient painting made from linseed oil with inorganic titanium white pigment particles was analyzed to identify the degradation of these materials with time. The characterization of oxidative products on the painting surface is found to be very useful for better understanding the degradation mechanisms and thus can be said to be the best method for better preserving these artworks [177].

The aging mechanism of a PET fiber under accelerated aging conditions is also reported [161]. Although, PET is a common polyester polymer and the aging mechanisms of this polymer have been extensively investigated. However, there are still open questions relating to the distribution of oxidized functional groups on the fiber surface. The homogeneity of the polymer degradation on the surface at the nanoscale and molecular structure changes during the aging process of PET, as well as the degradation mechanisms [161,163,179,180]. Nguyen-Tri et al. have employed the AFM-IR technique to detect the distribution of functional groups at the surface of the PET fiber before and after aging tests [161]. They could able to propose a complementary mechanism for the PET under accelerated aging conditions. In another case, AFM-IR is used to compare the mechanism of aging of an organic coating based on polyurethane in two different conditions as follows [166]: (i) in natural exposure up to 10 years, and (ii) in accelerated conditions. They analyzed the distribution of carbonyl groups on the surface of both naturally and artificially aged samples. Two aging mechanisms were observed. In the naturally exposed coating, the oxidized group developed from holes and crack borders to the bulk matrix and this process is inhomogeneous, while in the accelerated aging condition, the oxidized groups are homogeneously distributed on the whole surface without serious crack on the sample surface. From the kinetic data on properties and structural changes, the authors have compared the results in both cases and proposed models to calculate the service lifetime of these coating.

#### 3.3.2. AFM-IR in Biopolymers and Multilayers

Many bio-resourced based polymers have been synthesized or are formed in nature during the growth cycles of all organisms. The increase in the number of publications during recent years reflects the growing interest and importance of such new materials. The application of a new technique or method like AFM-IR to analyze morphology, properties and structure of these materials is attracting the more attention of the scientific community.

Mayet et al. [143] investigated the production of bacterial polyhydroxybutyrate in *Rhodobacter capsulatus* using TEM and AFM-IR. They concluded that AFM-IR was more useful and takes less time to prepare the sample compared to TEM. The samples can be ready for detailed analysis after 1 day of preparation instead of 2–3 weeks for TEM analysis. The other advantages of AFM-IR relate to its capacity to obtain chemical images to directly ascertain the presence of PHB in blends by analyzing AFM maps at the 1740 cm^−1^ band (carbonyl group) which the TEM imaging could not do. AFM-IR was also used to study a laminated multilayer film interface based on ethylene acrylic acid (EAA) copolymer/polyamide (nylon) blend. Compared to traditional FT-IR having a special resolution of around 10 μm, the use of AFM-IR shows better resolution at the interface. It was interesting to show that there was a very good correlation between the IR spectra of the polymer measured by bulk traditional FT-IR measurements and AFM-IR [55,171].

**Figure 11:**
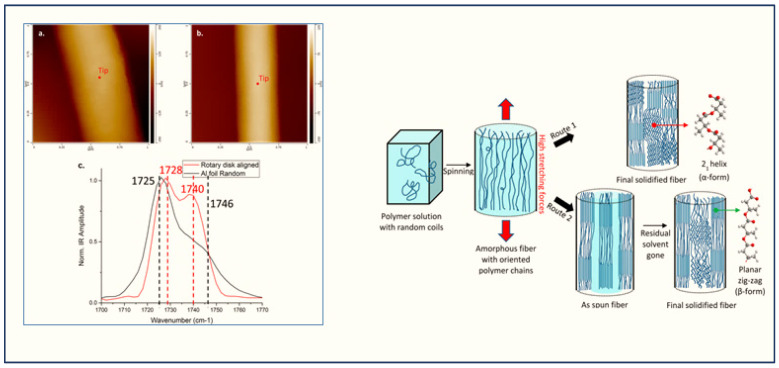
AFM-IR images and possible mechanisms for the formation of the α- and β-form crystal structure of single electrospun of PHBHx nanofibers. *Reproduced with copyright permission from Gong 2015* [56] (*American Chemical Society*).

Gong et al. [56] used the AFM-IR technique to study the structure and morphology of individual biodegradable fibers (Figure 11). They indicated that the IR peak in AFM-IR complements well with the traditional FT-IR spectra in terms of the position of the peak and their relative intensity. The spectra obtained from AFM-IR are highly resolved that that obtained from the traditional FT-IR. By using AFM-IR combined with other characterization techniques such as wide-angle X-ray diffraction (WAXD) and selected area electron diffraction (SAED), a new mechanism of β-form crystal structures in the interface has been reported (Figure 11). Recently, Kelchtermans et al. [64] have employed the AFM-IR technique for the characterization of polyethylene-polyamide (PE-PA) multilayer films. They also confirmed that, in thin tie layers at the scale level, the analysis of the chemical composition was readily obtained.

## 4. Summary and Outlooks

This paper provides the latest progress in the application of AFM-IR technique for polymer material studies dealing with polymer composites, polymer blends, biopolymers and multilayers. The paper demonstrated how this novel method can provide chemical information at the nanoscale scale based on the radiation absorption resulting from specific molecular vibrations. Secondly, we described the use of AFM and AFM-IR techniques to study the polymer crystallization in terms of the spherulite structure, lamella structure, and the distribution of polymer, by highlighting on two polymer blends of polyethylene glycol with polycaprolactone (PCL) (immiscible blend) and polyhydroxybutyrate (PHB) (miscible blend) at different scales ranging from inter-spherulite to inter-lamella. The paper discussed how AFM-IR can be used for investigating polymer aging and other systems including biopolymers and multilayer polymeric materials.

The recent applications found in the literature do not represent the real potential of this novel technique. It could certainly be applied in a broad range of research areas, including material science, polymer, composites, blends, 3D printing, pharmaceuticals, biomedical and life sciences. In the polymer crystallization investigation, the improvement of the spatial resolution of AFM-IR up to 10 nm could bring various additional advantages in analyzing the chemical composition of complex blends, especially for thin and ultrathin polymeric films. The availability of new devices for AFM-IR in terms of laser source and sample heating/cooling system will be addressed to investigate the crystallization in situ a larger number of polymeric systems including tertiary or multiphase blends and helps to better understand the crystallization of complex and miscible polymer blends at the nanoscale.
